# Antidepressant-Like Effects and Cognitive Enhancement of Coadministration of Chaihu Shugan San and Fluoxetine: Dependent on the BDNF-ERK-CREB Signaling Pathway in the Hippocampus and Frontal Cortex

**DOI:** 10.1155/2020/2794263

**Published:** 2020-02-22

**Authors:** Lijing Yan, Xia Xu, Zhenyu He, Sheng Wang, Linlin Zhao, Juan Qiu, Dongsheng Wang, Zhicheng Gong, Xinjian Qiu, Huiyong Huang

**Affiliations:** ^1^Laboratory of Ethnopharmacology, Institute of Integrated Traditional Chinese and Western Medicine, Xiangya Hospital, Central South University, Changsha, Hunan 410008, China; ^2^Taizhou Hospital of Integrated Traditional Chinese and Western Medicine, Taizhou, Zhejiang 317500, China; ^3^The Second Clinical Medical College, Zhejiang Chinese Medicine University, Hangzhou, Zhejiang 310053, China; ^4^Department of Health Management, The Third Xiangya Hospital of Central South University, Changsha, Hunan 410013, China; ^5^Department of Nuclear Medicine, The Third Xiangya Hospital of Central South University, Changsha, Hunan 410008, China; ^6^Department of Pharmacy, Institute for Rational and Safe Medication Practices, National Clinical Research Center for Geriatric Disorders, Xiangya Hospital, Central South University, Changsha, Hunan 410008, China; ^7^Institute of Traditional Chinese Medicine Diagnosis, Hunan University of Traditional Chinese Medicine, Changsha, Hunan 410208, China

## Abstract

**Background:**

Fluoxetine (FLU) is the first-line and widely used medication for depression; however, FLU treatment is almost ineffective in 30%-40% of patients with depression. In addition, there are some problems in FLU treatment, such as delayed efficacy, large side effects, and poor tolerance. Chaihu Shugan San (CSS) is a classic and effective antidepressant Chinese herbal medicine that has been used in China for thousands of years. CSS or coadministration of CSS and FLU has become one of the most recommended methods in the treatment of depression in China. However, the specific pathways of CSS and coadministration of CSS and FLU for antidepressant are still unclear.

**Objective:**

This study was designed to evaluate the antidepressant effects of CSS and coadministration of CSS and FLU.

**Methods:**

The chronic unpredictable mild stress (CUMS) rat model was used to simulate depression. 120 healthy adult male Sprague-Dawley (SD) rats were randomly divided into seven groups: the control group, CUMS group, low-dose CSS group, high-dose CSS group, FLU group, coadministration of low-dose CSS and FLU group, and coadministration of high-dose CSS and FLU group. The rats in different groups were given different interventions. Then, the depression-like behavior and cognitive function were evaluated by the sucrose preference test (SPT), forced swimming test (FST), open field test (OFT), and Y-maze test. What is more, the antidepressant mechanism of CSS and coadministration of CSS and FLU were studied through BDNF mRNA, ERK mRNA, CREB mRNA, BDNF, p-ERK/ERK, and p-CREB/CREB levels in the hippocampus and frontal cortex by Western blot and RT-PCR.

**Results:**

Compared with the CUMS group, CSS and coadministration of CSS and FLU could alleviate the depressive symptoms and improve cognitive function in CUMS rats (*p* < 0.05); CSS and coadministration of CSS and FLU could increase the expression of BDNF, p-CREB/CREB, p-ERK/ERK, and BDNF mRNA, CREB mRNA, and ERK mRNA in the hippocampus and frontal cortex (*p* < 0.05); CSS and coadministration of CSS and FLU could increase the expression of BDNF, p-CREB/CREB, p-ERK/ERK, and BDNF mRNA, CREB mRNA, and ERK mRNA in the hippocampus and frontal cortex (*p* < 0.05); CSS and coadministration of CSS and FLU could increase the expression of BDNF, p-CREB/CREB, p-ERK/ERK, and BDNF mRNA, CREB mRNA, and ERK mRNA in the hippocampus and frontal cortex (*Discussion and Conclusion*. Finally, we found that both CSS and coadministration of CSS and FLU play an antidepressant role, which may be due to the regulation of the BDNF/ERK/CREB signaling pathway in the hippocampus and frontal cortex. Among them, the coadministration of CSS and FLU can enhance the antidepressant effect of CSS or FLU alone, and the underlying mechanism needs further investigation.

## 1. Introduction

Depression is a mood disorder characterized by persistent feeling of sadness, loss of interest, decline in thinking and cognitive function, and disorder of physiological function [1, 2]. The WHO reported that it could be the third principal cause of disability worldwide by 2020 [3]. In addition, depression can easily lead to suicide and decreased fertility [4–6]. Based on data from the 2012 China Family Panel Studies survey, studies have shown that mental illness contributes to 14.7% of total personal expected medical spending in China, respectively, with depression and depressive symptoms accounting for 6.9% and 7.8% [7]. Therefore, it is not difficult to conclude that depression is a major neurological disease that seriously threatens human health and quality of life. Depression causes a serious burden to the society, and there is an urgent need for safe and effective treatment [8].

At present, common antidepressants mainly include tricyclic antidepressants and selective serotonin (5-HT) reuptake inhibitors. However, tricyclic antidepressants have adverse effects on the liver, kidneys, and heart according to the related report [9]. Fluoxetine, a selective serotonin (5-HT) reuptake inhibitor, is the first-line and widely used medication for depression; however, fluoxetine treatment is almost ineffective in 30%-40% of patients with depression. In addition, the treatment with fluoxetine has some problems such as delayed effect, large side effects, and poor tolerance [10, 11]. For example, it can cause severe side effects such as fatigue, headache, loss of appetite, weight gain, nausea, and bad mood [12]. Objective or subjective serious side effects usually force some patients to abandon medication [13]. Considering the possible harm, the lack of effective and safe treatments worldwide, and the high incidence of depression, more effective and safe antidepressants are urgently needed. Phytochemicals and medicinal herbs could be a dependable alternative antidepressant for those patients who do not obtain benefit from conventional antidepressant [14].

It is estimated that up to 80% of the population in developing countries use traditional herbs for primary health care [15]. CSS or coadministration of CSS and FLU is commonly used to enhance antidepressant effect and reduce side effects in Chinese clinics [16], which was confirmed by multiple studies [17–19]. Chaihu Shugan San (CSS) is a classic and effective antidepressant Chinese herbal medicine that has been used in China for thousands of years. CSS or coadministration of CSS and FLU has become one of the most recommended methods for the treatment of depression in China.

CSS consists of seven Chinese herbs, i.e., the root of Bupleurum chinense DC. (Chai-hu), the root of Paeonia lactiflora Pall. (Bai-shao), the pericarps of Citrus reticulata Blanco (Chen-pi), the root of Ligusticum chuanxiong Hort. (Chuan-xiong), the root of Cyperus rotundus L. (Xiang-fu), the fruit of Citrus aurantium L. (Zhi-qiao), and the root of Glycyrrhiza uralensis Fisch. (Gan-cao) [20, 21]. Our previous study found that chronic unpredictable mild stress (CUMS) affects neuroendocrine, behavior, and related molecular expression and ultimately leads to depression in rats. CSS can regulate the behavior of CUMS rats by regulating JNK and ERK1/2 mRNA expression in the hippocampus [22–24]. Related reports have found that CSS can significantly improve the depression status of model rats, and its mechanism may be related to the increase of mRNA expression of BDNF and TrkB in the hippocampus, amygdala, and frontal lobe [25].

Fluoxetine can increase the occurrence of hippocampal neurons in rats with depression, improve the stereology of synaptic structures, and restore the structure and function of the hippocampus [26]. Fluoxetine can reduce the expression of Bax mRNA in the hippocampus of rats with depression, increase the expression of Bcl-2 mRNA, and reduce neuronal apoptosis [27]. In summary, neuronal regeneration and apoptosis, neuroplasticity, BDNF, and related pathway regulation may be a common mechanism of antidepressants such as CSS and fluoxetine.

The combination of CSS and fluoxetine can effectively alleviate the symptoms of patients with depression. However, the potential neuroprotective effects and mechanisms of CSS and fluoxetine on CUMS-induced depression remain to be elucidated. Therefore, in this study, we used the CUMS model to study the antidepressant-like effects and cognitive enhancement of coadministration of CSS and fluoxetine. In addition, BDNF mRNA, ERK mRNA, CREB mRNA, BDNF, p-ERK/ERK, and p-CREB/CREB levels in the hippocampus and frontal cortex were measured to explain the possible mechanism of CSS and fluoxetine.

## 2. Materials and Methods

### 2.1. Experimental Animals

120 male clean healthy adult SD rats (body weight 200 ± 20 g) were provided by the Animal Experimental Center of Central South University (Hunan, China), and the experimental animal license number was SYXK (Xiang) 2016-0301. The mice were housed individually, lit at 07:00 am, then given light on a schedule of 12-hour light and 12-hour dark followed. The feeding space is maintained at an ambient temperature of 23-25°C and a relative humidity of 54%-66%. Throughout the experiment, animals were given food and water unless otherwise stated. All procedures were approved and implemented in accordance with the guidelines of the ethics of Xiangya Hospital in Central South University.

### 2.2. CSS

The CSS formula used in this experiment is based on our previous research [13]. All Chinese medicines were purchased by the Chinese Pharmacy of Xiangya Hospital and identified by the Associate Professor Lei Peng in compliance with the Pharmacopoeia requirements. The origin, batch number, and ratio of the herb medicine are displayed in Table 1.

### 2.3. Drugs and Reagents

20 mg/granules fluoxetine hydrochloride capsules were purchased from Lilly Suzhou Pharmaceutical Co., Ltd. Rabbit anti-mouse ERK1/2 antibody was purchased from the American Abcam company. Rabbit anti-mouse BDNF antibody was purchased from Abcam, USA. The PCR reverse transcription kit was purchased from Promega. Rabbit anti-mouse pERK1/2 antibody was purchased from Abcam, USA. The normal goat serum for closure was purchased from the American Abcam company. The streptavidin-biotin-horseradish peroxidase complex was purchased from VECTOR Bio GmbH, USA. The SABC kit was purchased from VECTOR Bio GmbH, USA. The BCA protein quantification kit was purchased from the American Abcam company. TRIzol was purchased from the Invitrogen company. Chloroform, isopropanol, and absolute ethanol were of analytical grade and were purchased from Shanghai Chemical Reagent Company. DNA marker was purchased from Beijing Dingguo Biotechnology Company.

### 2.4. Preparation of CSS Extract

The original herbal ingredients of CSS were mixed and crushed into small pieces in a ratio of 4 : 4 : 3 : 3 : 3 : 3 : 1. The compound was immersed in water (1 : 8, *w*/*v*) for 30 minutes at room temperature, then heated to boiling, and boiled for a further 0.5 hours. The filtrate was collected, and the residue was refluxed in the same volume of water and heated for a further 0.5 hours. The filtrate was collected, and the residue was refluxed in the same volume of water and heated for a further 0.5 hours. The two filtrates were combined and concentrated in vacuo to give a CSS extract of 2.1 g/mL. When used, it is made into a certain concentration with distilled water as needed [11].

### 2.5. Drug Administration

To study the antidepressant effects of CSS and fluoxetine, rats were randomly divided into the following 7 groups (*n* = 15) using a random number table: control group (saline), model group (saline), low-dose CSS group (CSS 5.9 g/(kg·d)), high-dose CSS group (CSS 11.8 g/(kg·d)), fluoxetine group (FLU 1.8 g/(kg·d)), coadministration of low-dose CSS and FLU group (CSS 5.9 g/(kg·d)+FLU 1.8 g/(kg·d)), coadministration of high-dose CSS and FLU group (CSS 11.8 g/(kg·d)+FLU 1.8 g/(kg·d)). The chronic unpredictable mild stress (CUMS) rat model was used to simulate depression. Except the control group, the other six groups received unpredictable mild stress for 28 days to simulate depression. The rats in different groups were given different interventions for 4 weeks. The combination groups were given fluoxetine one hour after the administration of CSS. Behavioral tests were performed before and after the administration.

### 2.6. CUMS Procedure

We established chronic depression models with chronic unpredictable mild stress. CUMS includes exposure to various unpredictable stress factors (random), including fasting (24 h), 4°C cold water swimming (5 min), tail (1 min), 45°C hot water swimming (5 min), restraint (3 h), shaking (60 times/min, 10 min), and moist litter (24 h).

A total of 7 kinds of stimulation methods were randomly stimulated in 28 days to stimulate the rats. In order to avoid the rats being accustomed to the same kind of stimulation, the same stimulation was not performed for two consecutive days. All pressures were applied individually and continuously during the day and night, and each stimulus was used 2-3 times cumulatively. Rats in the control group remained undisturbed at all times, except for necessary procedures such as routine cage cleaning.

### 2.7. Behavioral Testing

#### 2.7.1. Sucrose Preference Test (SPT)

Rats were trained for 72 hours before the start of the experiment. The first 24 hours gave rats 2 bottles of 1% syrup, and the second 24 hours gave rats 1% syrup and 1 bottle of pure water. The third 24 hours deprived rats of food and water. Then, a total of 2 bottles of syrup and pure water (100 mL each) were administered to each rat, and the consumption of 1% syrup and pure water was measured for 1 hour to calculate the saccharide consumption rate: sugar consumption rate = sugar water consumption/sugar water + pure water consumption.

#### 2.7.2. Open Field Test (OFT)

Using a 100 cm × 100 cm × 40 cm open box device, the bottom of the box was divided into 25 equal squares and the rats were quickly placed in the center of the box. Calculate the number of crossings in 5 minutes (horizontal motion score: the score of the four-claw into the bottom square is 1 point) and the number of hind legs upright (vertical exercise score: the number of uprights, that is, the two front paws vacated off the ground or climbed the four walls as the scoring standard, 1 score 1 time).

#### 2.7.3. Forced Swimming Test (FST)

The test was carried out by placing the rat in a glass cylinder (46 cm high, 20 cm diameter) filled with 30 cm high water (25 ± 2°C). Two swim trainings were performed: an initial 15-minute pretest and then a 5-minute trial after 24 hours. As long as the mouse floats passively in the water and only makes a slight movement to keep its head above the waterline, it is considered to be stationary. What is more, we changed water between each test. The test session scored the immobile time by a qualified observer who turned a blind eye to treatment.

#### 2.7.4. The Y-Maze Test

The Y-maze test is used as a measure of real-time spatial working memory to measure short-term memory. The Y-shaped labyrinth consists of three arms of equal angle (30 cm long × 5 cm wide × 12 cm high). Place the mouse at the end of one arm and allow it to move freely for 6 minutes in the maze. When the mouse's hind paw is completely inside the arm, the arm is counted. Visually record a series of arm entries and calculate the percentage change. Spontaneous alternation is defined as the continuous entry into three arms, namely, ABC, CAB, or BCA but not CBC. The percentage is calculated as the ratio of actual alternation to possible alternation (defined as the total number of arm entries minus 2) multiplied by 100, as shown in the following equation: alternate% = [number of alternations/(total number of arms − 2)] × 100. The number of arm inlets is also used as an indicator of athletic activity.

### 2.8. Western Blot Assay

The frontal cortex and hippocampus were isolated from the rat brains and homogenized in lysis buffer (50 mm Tris–Cl, 150 mm NaCl, 0.02% NaN2, 100 *μ*g/mL phenylmethanesulfonyl fluoride, 1 *μ*g/mL aprotinin, and 1% Triton X-100) in the presence of a protease inhibitor and phosphatase inhibitor on ice. The lysate was centrifuged at 13,000 rpm for 10 min to obtain the supernatant. The protein concentrations were determined by the BCA protein assay kit. The lysed samples were separated by SDS-PAGE and then transferred to polyvinylidene fluoride (PVDF) membranes. After blocking with 5% skim milk for 2 h at room temperature, the membranes were incubated with specific primary antibodies at 4°C overnight. The PVDF membranes were then incubated with HRP-conjugated secondary antibodies for 2 h, and protein bands were captured via enhanced chemiluminescence. The gray intensity of the protein bands was analyzed by ImageJ software (NIH, Bethesda, MD).

### 2.9. RT-PCR

Total RNA was extracted and isolated from the hippocampus and cerebral cortex. Next, the concentration and purity of RNA were measured. Then, the high-quality total RNA was reverse transcribed. The primers of the target genes and GAPDH were used, as described as follows: BDNF: 5′-AGCTTGTATCCGACCCTCTCTG-3′ and R: 5′-CAGCAATCAGTTTGTTCGGC-3′; CREB: 5′-CTGATTCCCAAAAACGAA-3′ and R: 5′-CTGCCCACTGCTAGTTTGGT-3′; ERK: 5′-TACCGAGCCCCAGAGATCAT-3′ and R: 5′-GGAAGATAGGCCGGTTGGAG-3′; and GADPH: 5′-CCATGTTTGTGATGGGTGTG-3′ and R: 5′-CCTTCCACAATGCCAAAGTT-3′. Real-time quantitative PCR analysis was performed with a SYBR® Premix Ex Taq. The relative expression levels of target genes were normalized against the level of GAPDH in the same cDNA by using the relative quantification method (2−*ΔΔ*CT).

### 2.10. Statistical Analyses

Data were analyzed using the Statistical Program for the Social Sciences statistical software (SPSS 16.0, NY, USA). All data in the present study are expressed as the mean ± SEM. The data were first analyzed with Student's *t*-test or one-way ANOVA, followed by Tukey's post hoc test. A value of *p* < 0.05 was considered to be statistically significant for analysis.

## 3. Results

### 3.1. CSS and Coadministration of CSS and FLU Alleviate the Depressive Symptoms of Depression, Evasion, and Despair in CUMS Rats

**Figure 1:**
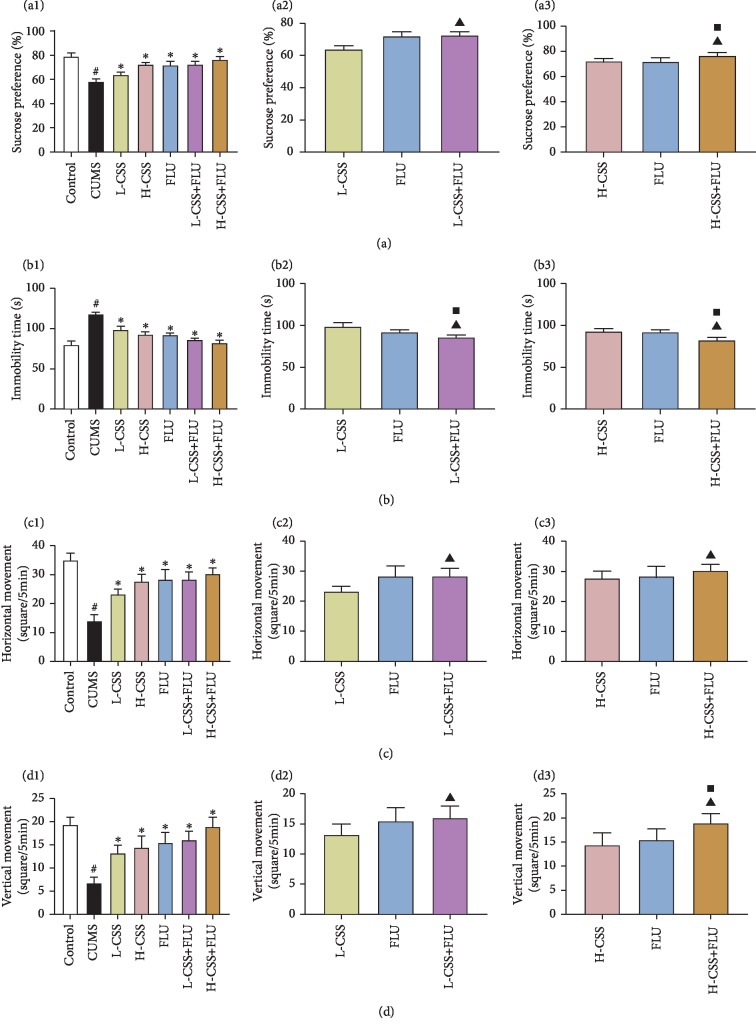
CSS and coadministration of CSS and FLU improve depression, evasion, and despair in CUMS rats. The difference *p* < 0.05 was considered to be statistically significant. The syrup preference index of the sucrose preference test (SPT) (a). The immobility time of the forced swimming test (FST) (b). The horizontal movement (c) and vertical movement (d) of the open field test (OFT). Compared with the control group, ^#^*p* < 0.05; compared with the CUMS group, ^∗^*p* < 0.05; compared with the CSS group, ^▲^*p* < 0.05; compared with the FLU group, ^■^*p* < 0.05.

After different interventions, compared with the control group, the syrup preference index in the sucrose preference test (SPT) and the horizontal and vertical movements in the open field test (OFT) in the CUMS group were significantly decreased (*p* < 0.05), and the immobility time in the forced swimming test (FST) was significantly increased (*p* < 0.05). Compared with the CUMS group, the sucrose preference index and the horizontal and vertical movements of the different intervention groups were significantly increased (*p* < 0.05), and the immobility time was significantly decreased (*p* < 0.05), suggesting that CSS and coadministration of CSS and FLU both could alleviate the depressive symptoms of depression, evasion, and despair in CUMS rats. Among them, we can learn that high-dose CSS and CSS combined with fluoxetine can better alleviate depressive symptoms. In addition, we found that the sucrose preference index, the horizontal and vertical movements, and the immobility time of the coadministration of CSS and FLU were significantly better than CSS alone (*p* < 0.05) and fluoxetine alone (*p* < 0.05), suggesting that coadministration of CSS and FLU could better alleviate the depressive symptoms of depression, evasion, and despair in CUMS rats whether it is administered with CSS alone or fluoxetine alone (Figure 1).

### 3.2. CSS and Coadministration of CSS and FLU Improve Cognitive Function in CUMS Rats

**Figure 2:**
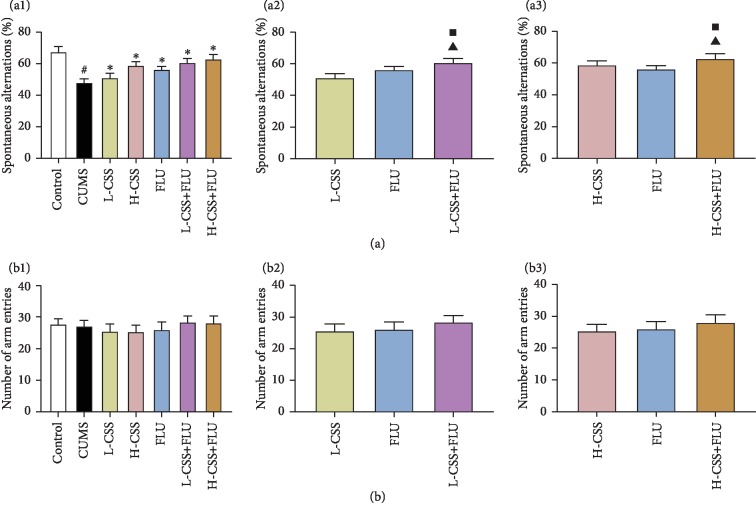
CSS and coadministration of CSS and FLU improve cognitive function in CUMS rats. The difference *p* < 0.05 was considered to be statistically significant. The spontaneous alternation (a) and arm entries (b) in the Y-maze test. Compared with the control group, ^#^*p* < 0.05; compared with the CUMS group, ^∗^*p* < 0.05; compared with the CSS group, ^▲^*p* < 0.05; compared with the FLU group, ^■^*p* < 0.05.

After different interventions, there was no significant difference in the number of arm entries in the Y-maze test between the groups (*p* > 0.05), indicating that the percentage of spontaneous alternation in each group was not related to the change of exercise activity. Compared with the control group, the percentage of spontaneous alternation in the Y-maze test in the CUMS group was significantly decreased (*p* < 0.05). Compared with the CUMS group, the percentage of spontaneous alternation of the different intervention groups was significantly increased (*p* < 0.05), suggesting that CSS and coadministration of CSS and FLU both could improve cognitive function in CUMS rats. Among them, we can see that high-dose CSS and CSS combined with fluoxetine can better improve the cognitive function. What is more, we found that the percentage of spontaneous alternation of the coadministration of CSS and FLU was significantly better than CSS alone (*p* < 0.05) and fluoxetine alone (*p* < 0.05), suggesting that coadministration of CSS and FLU could better improve cognitive function in CUMS rats whether it is administered with CSS alone or fluoxetine alone (Figure 2).

### 3.3. CSS and Coadministration of CSS and FLU Regulate the ERK-CREB-BDNF Signaling Pathway in the Hippocampus and Frontal Cortex

**Figure 3:**
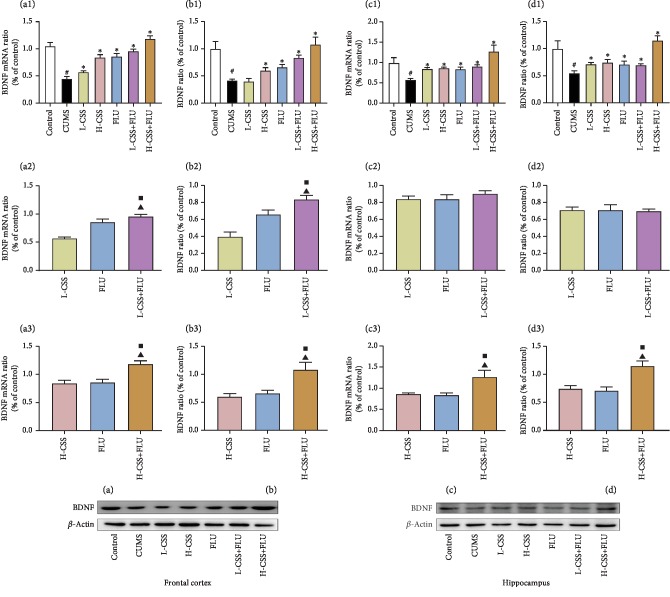
CSS and coadministration of CSS and FLU improve the expression of BDNF mRNA and protein in the frontal cortex and hippocampus of CUMS rats. The difference *p* < 0.05 was considered to be statistically significant. BDNF mRNA in the frontal cortex (a) and hippocampus (c). BDNF protein expression in the frontal cortex (b) and hippocampus (d). Compared with the control group, ^#^*p* < 0.05; compared with the CUMS group, ^∗^*p* < 0.05; compared with the CSS group, ^▲^*p* < 0.05; compared with the FLU group, ^■^*p* < 0.05.

**Figure 4:**
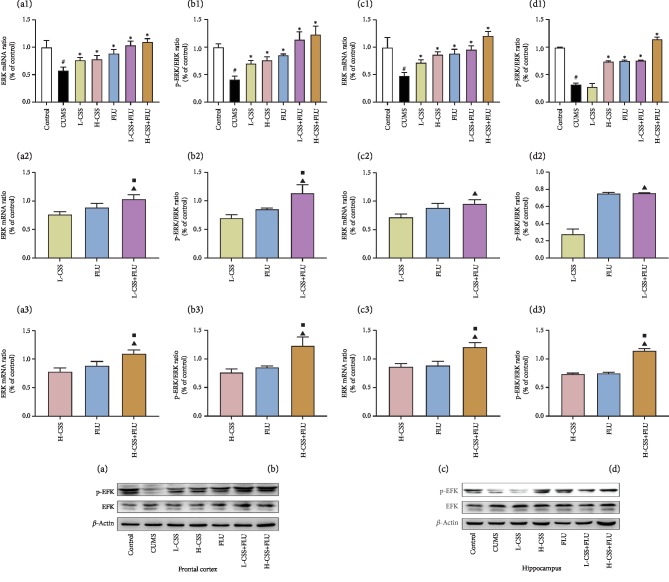
CSS and coadministration of CSS and FLU improve the expression of ERK mRNA and p-ERK/ERK in the frontal cortex and hippocampus of CUMS rats. The difference *p* < 0.05 was considered to be statistically significant. ERK mRNA in the frontal cortex (a) and hippocampus (c). p-ERK/ERK in the frontal cortex (b) and hippocampus (d). Compared with the control group, ^#^*p* < 0.05; compared with the CUMS group, ^∗^*p* < 0.05; compared with the CSS group, ^▲^*p* < 0.05; compared with the FLU group, ^■^*p* < 0.05.

**Figure 5:**
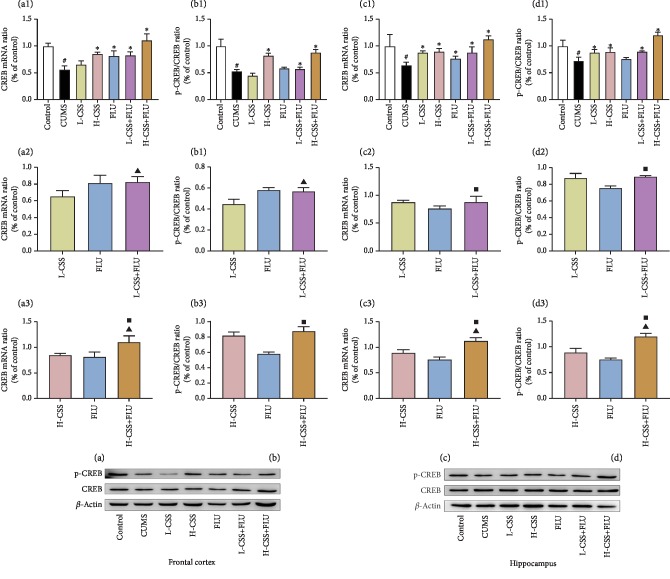
CSS and coadministration of CSS and FLU improve the expression of CREB mRNA and p-CREB/CREB in the frontal cortex and hippocampus of CUMS rats. The difference *p* < 0.05 was considered to be statistically significant. CREB mRNA in the frontal cortex (a) and hippocampus (c). p-CREB/CREB in the frontal cortex (b) and hippocampus (d). Compared with the control group, ^#^*p* < 0.05; compared with the CUMS group, ^∗^*p* < 0.05; compared with the CSS group, ^▲^*p* < 0.05; compared with the FLU group, ^■^*p* < 0.05.

After different interventions, the expression of BDNF mRNA, CREB mRNA, ERK mRNA, BDNF, p-CREB/CREB, and p-ERK/ERK in the frontal cortex and hippocampus of the CUMS group were significantly lower than the control group (*p* < 0.01). Compared with the CUMS group, high-dose CSS and coadministration of high-dose CSS and FLU could increase the expression of BDNF, p-CREB/CREB, p-ERK/ERK, and BDNF mRNA, CREB mRNA, and ERK mRNA in the hippocampus and frontal cortex (*p* < 0.05). Low dose of CSS promoted the expression of BDNF mRNA, p-ERK/ERK, and ERK mRNA in the frontal cortex (*p* < 0.05) and the expression of BDNF, BDNF mRNA, p-CREB/CREB, CREB mRNA, p-ERK/ERK, and ERK mRNA in the hippocampus (*p* < 0.05). The coadministration of high-dose CSS and FLU promoted the expression of BDNF, p-ERK/ERK, BDNF mRNA, CREB mRNA, and ERK mRNA in the frontal cortex and hippocampus of the CUMS group (*p* < 0.05) and the expression of p-CREB/CREB in the hippocampus (*p* < 0.05). In addition, we found that the expression of BDNF mRNA, BDNF, p-CREB/CREB, CREB mRNA, p-ERK/ERK, and ERK mRNA in the frontal cortex of the coadministration of high dose of CSS and FLU was significantly better than CSS alone (*p* < 0.05) and fluoxetine alone (*p* < 0.05), suggesting that CSS can promote the expression of ERK mRNA, p-ERK/ERK, CREB mRNA, p-CREB/CREB, BDNF mRNA, and BDNF in the hippocampus and frontal cortex. CSS regulates the BDNF-ERK-CREB signaling pathway to play an antidepressant-like role, while the coadministration of CSS and FLU potentiates this regulation (Figures 3, 4, and 5).

## 4. Discussion

In this study, the CUMS rat model was used to study the antidepressant-like effects of CSS and fluoxetine. The depression-like behavior and cognitive function were evaluated by the sucrose preference test (SPT), forced swimming test (FST), open field test (OFT), and Y-maze test. What is more, the antidepressant mechanism of CSS and coadministration of CSS and FLU were studied through BDNF mRNA, ERK mRNA, CREB mRNA, BDNF, p-ERK/ERK, and p-CREB/CREB levels in the rat hippocampus and frontal cortex by Western blot and RT-PCR.

Many studies have found that mental illness often manifests as a significant cognitive impairment, and the assessment of cognitive function plays an important role in assessing mental illness [28, 29]. Chronic unpredictable mild stress (CUMS) is currently the most commonly used and most convincing rodent model of depression [30], and studies have shown that the CUMS program is a robust animal model of depression [31]. Depression-like behavior in rats can be reasonably inferred by using appropriate animal models and behavioral tests including the sucrose preference test (SPT), forced swimming test (FST), Y-maze test, and open field test [32]. Among them, the sucrose preference index in the sucrose preference test (SPT) was used to assess the loss of pleasure, the alternating percentage of the Y-maze test was used to assess the brain cognitive function, the forced time of the forced swimming test (FST) was used to evaluate the depression in animal models, and the horizontal and vertical movements in the open field test (OFT) was used to assess the animal evasion [33–35]. Our study found that CUMS can reduce the sucrose preference index, horizontal movement, vertical movement activity, and alternating percentage and increase the duration of immobility. In contrast, the sucrose preference index, horizontal movement, vertical movement, and percentage of alternation can be increased by the intervention of CSS; the duration of immobility, relieving depression, and cognitive dysfunction in rats can be reduced by it. The sucrose preference index, horizontal movement, vertical movement, alternating percentage, and immobility status of CSS combined with the fluoxetine group were better than their corresponding CSS and fluoxetine groups. Among them, in each test, the combination treatment group was significantly better than the CSS group (*p* < 0.01), and the high-dose CSS combined with the fluoxetine group was significantly better than the fluoxetine group (*p* < 0.01). CSS and CSS combined with FLU improved the memory formation, despair, and pleasure of CUMS.

At present, considerable progress has been made in understanding the pathophysiology of severe depression, but there is still no model or mechanism that can be satisfactorily explained from all aspects [8]. In recent years, with the rapid development of neurophysiology and neuropathology, people gradually realize that depression is a complex physiological dysfunction caused by different reasons [36–38]. There are many hypotheses about the pathogenesis of depression, and neurotrophic factor deficiency is one of the most important hypotheses [39, 40]. BDNF can be expressed in various brain regions such as the cerebral cortex and hippocampus, which has biological functions to maintain synaptic growth as well as the growth, differentiation, and survival of neurons [41]. The BDNF transcription and regulation mechanisms are very complex as the multiple transcripts and complex transcriptional regulatory machinery [42]. A number of studies have demonstrated that BDNF preferentially binds to the TrkB receptor, and dysfunction of the BDNF/TrkB system is associated with the pathophysiology of brain diseases, including neurodegenerative diseases and mental illness [43–45]. In addition, various studies have shown that BDNF activates the MAPK cascade after binding to TrkB, of which the ERK pathway is one of the best characterized signal transduction pathways [46, 47]. The ERK pathway runs through the cell membrane and the nucleus, which effectively connects external signals with intracellular signals [48, 49]. CREB is a transcription factor and a downstream target of ERK [50, 51]. A series of studies have revealed the important role of the BDNF-ERK-CREB signaling pathway in depression-like behavior and antidepressant effects. Recent clinical studies have shown that BDNF is a promising biomarker, and BDNF levels in serum of patients with depression are significantly reduced, while antidepressant treatment reverses this effect [52, 53]. Deleting BDNF in broad forebrain regions reduces the brain ability of learning and memory and greatly reduces the antidepressant ability of the antidepressant desipramine [54]. In a stressful environment, variant BDNF mice (BDNF (Met/Met)) showed anxiety, and this anxiety could not be reversed by fluoxetine [55]. Depletion of BDNF by transfection of lentiviral-derived shBDNF in the hippocampus inhibits the antidepressant effect of fluoxetine [56]. Chronic peripheral administration of BDNF can increase BDNF, p-ERK, and p-CREB levels in the hippocampus of mice, playing an antidepressant effect by directly increasing BDNF levels in the brain or activating TrkB-ERK-CREB signaling possibly [57–59]. Inhibitors of the ERK pathway include U0126 and specific MEK inhibitors. In order to provide direct evidence of the potential role of the ERK pathway in depression, mice were infused with U0126 infusion into the dorsal hippocampus (dHP) and prefrontal cortex (mPFC), after which the depressive symptoms including lack of pleasure and anxiety-like behavior appear and the p-CREB levels of dHP and mPFC decreased [49]. In conclusion, these results indicate that BDNF is an important biomarker of depression and may exert antidepressant effects by activating ERK and CREB. Our data suggest that CSS can improve depression and cognitive function in CUMS rats and the coadministration of CSS and FLU enhances the antidepressant effects. CSS and coadministration of CSS and FLU may increase the level of CREB transcription and protein phosphorylation in the hippocampus and frontal cortex by increasing ERK transcription and protein phosphorylation in the hippocampus and frontal cortex, ultimately affecting BDNF transcriptional levels and increasing BDNF protein levels in the hippocampus and frontal cortex, which may regulate the ERK-CREB-BDNF pathway and play an antidepressant role as a result. In addition, CSS antidepressant effect is dose-dependent, and CSS has a dose-dependent effect on the transcription and expression of BDNF and CERB.

Besides, our results found that the coadministration of high dose of CSS and FLU is superior to high-dose CSS or fluoxetine in improving the depressive symptoms and cognitive function of CUMS rats. Previous studies have found that CSS can affect the pharmacokinetics of fluoxetine [60]. However, the mechanism of this enhancement is still unknown and the underlying mechanism needs further investigation.

## 5. Conclusions

Taken together, we found that both CSS and coadministration of CSS and FLU can relieve and improve the depression-like behavior and cognitive function, which may be due to the regulation of the BDNF/ERK/CREB signaling pathway in the hippocampus and frontal cortex. Among them, the coadministration of CSS and FLU can enhance the antidepressant effect of CSS or FLU alone, and there may be the drug interaction between CSS and FLU. The underlying mechanism needs further investigation.

## Figures and Tables

**Table 1:** The recipe, origin, and batch number of CSS.

Herb medicine	Origin	Batch number	Ratio
Bupleurum chinese DC. (Chai-hu)	Hebei Province	201606353	4
Pericarpium citri reticulatae (Chen-pi)	Jiangxi Province	201610342	4
Ligusticum chuanxiong Hort. (Chuan-xiong)	Sichuan Province	201610145	3
Rhizoma cyperi (Xiang-fu)	Hunan Province	201606010	3
Fructus aurantii (Zhi-ke)	Jiangxi Province	201607345	3
Radix paeoniae alba (Shao-yao)	Zhejiang Province	201608435	3
Glycyrrhiza uralensis Fisch. (Gan-cao)	Inner Mongolia	201608045	1

## Data Availability

The data used to support the findings of this study are available from the corresponding authors upon request.
